# Collective strategies to cope with work related stress among nurses in resource constrained settings: An ethnography of neonatal nursing in Kenya

**DOI:** 10.1016/j.socscimed.2019.112698

**Published:** 2020-01

**Authors:** Jacob McKnight, Jacinta Nzinga, Joyline Jepkosgei, Mike English

**Affiliations:** a Nuffield Department of Medicine, University of Oxford, Oxford, UK; b KEMRI-Wellcome Trust Research Programme, Nairobi, Kenya

**Keywords:** Kenya, Nursing, Coping, Burnout, Collective strategies, Resilience, Stress, Anxiety

## Abstract

Kenyan neonatal nurses are asked to do the impossible: to bridge the gap between international standards of nursing and the circumstances they face each day. They work long hours with little supervision in ill-designed wards, staffed by far too few nurses given the pressing need. Despite these conditions, a single neonatal nurse can be tasked with looking after forty sick babies for whom very close care is a necessity. Our 18-month ethnography explores this uniquely stressful environment in order to understand how nurses operate under such pressures and what techniques they use to organise work and cope. Beginning in January 2015, we conducted 250 h of non-participant observation and 32 semi-structured interviews in three newborn units in Nairobi to describe how nurses categorise babies, balance work across shifts, use routinised care, and demonstrate pragmatism and flexibility in their dealings with each other in order to reduce stress. In so doing, we present an empirically based model of the ways in which nurses cope in a lower-middle income setting and develop early work in nursing studies that highlighted collective strategies for reducing anxiety. This allows us to address the gap left by prevalent theories of nursing stress that have focused on the personal characteristics of individual nurses. Finally, we extend outwards from our ethnographic findings to consider how a deeper understanding of these collective strategies to reduce stress might inform policy, and why, even when the forces that create stress are alleviated, the underlying model of nursing work may prevail.

## Highlights

• Describes the understudied environment of LMIC inpatient newborn wards.
• Details how stress is managed through the design and enactment of nursing work.
• Develops and extends early theories of nurses' collective coping methods.
• Offers an alternative to questionnaire-based understandings of nursing stress.
• Links low-resource environments to nurse coping mechanisms.

## 1 Introduction

Nursing is central to the provision of hospital-based care (Hughes, 2006; Wilson et al., 2012) and is particularly so in the treatment of newborns (Dickson et al., 2014; Gathara et al., 2011). Continuous, effective provision of a basic set of interventions can have a highly positive impact on neonatal mortality, and most of these key interventions are delivered by nurses (Bhutta et al., 2014; Enweronu-Laryea et al., 2015).

Unfortunately, neonatal wards in low income settings are typified by a high ratio of sick infants to nurses (Aluvaala et al., 2015), which makes it difficult to deliver even basic care (Vesel et al., 2013) and limits the level of quality that is achievable (Kruk et al., 2018). In the context of neonatal nursing in low-income countries, nursing stress is of particular concern because workloads are higher and the demands on individuals are greater.

A deep understanding of this context is highly relevant to efforts directed towards the Sustainable Development Goals. SDG 3.2 renews efforts to reduce child mortality (WHO, 2017). Lawn et al. (2014) show that as much as 40% of all child deaths may be accounted for by neonatal mortality and recent research suggests that inpatient neonatal care is key to survival (Moxon et al., 2015). Human Resources for Health (HRH) are seen as the major limiting factor in addressing this need (Enweronu-Laryea et al., 2015; Moxon et al., 2015).

In the course of our study, it became clear that nurses were simply unable to meet national or international guidelines of best practice (e.g. WHO, 2016) and that asking nurses to translate such high aims into practice is unreasonable (Walker and Gilson, 2004). We are not the first to point to the stark contrast between what nurses are expected to do and what they actually do (Allen, 2004, 2007), but there has been little empirical work based in wards similar to the Kenyan New Born Units we study. We do not wish to assess this space purely in terms of underperformance relative to foreign guidelines, but instead to understand nursing practice within this particularly stressful context (Duclos et al., 2019). The sheer volume of tasks Kenyan neonatal nurses are faced with (Murphy et al., 2018b) means they must make stark choices about where to deploy their time and resources and we argue that this context provides an opportunity to extend theories of nursing overwork and coping.

While a great deal of research has been directed towards nursing stress, the study of how stress affects nursing practice at the ward level has not been a priority, particularly in LMIC settings. Instead, the study of nursing over-work, burnout and resilience has largely been focused on individuals and their personal, psychological characteristics. In the course of our study, we found that theories of individualised burnout and resilience did not help to explain the practices that seemed most important in reducing nurses' exposure to stress. Thus, our research question asks how nurses collectively cope with workload and stress and how this affects nursing practice.

### 1.2 Nursing practice and nursing stress in low-income hospitals

Hospitals tend to reflect and contribute to the local cultures in which they are embedded (Van Der Geest and Finkler, 2004). This seems particularly apparent for hospitals in Lower and Middle Income (LMIC) settings where practice may be quite far removed from idealised clinical methods. For example, d'Alessandro (2015) explores infection control in West African hospitals and finds that there is a great deal of mixing of clinical and social practices. d'Alessandro concludes that in this context, infection control is extremely hard and unlikely to be effective. Similarly, Strong (2017) investigates the effects of inadequate budgets on medical practice in a Tanzanian hospital. Strong (2017, p217) describes how this ‘environment of scarcity’ resulted in attempts to find workarounds by medical staff, but also pervasive low morale that is born of the stresses associated with low resources and persists even in better financed periods. While a lack of resources is key to understanding LMIC contexts, Fassin (2008) also shows that organisational and professional norms drive behaviour and that these more structural factors can themselves be linked to historical inequalities in society rather than being rooted in simple resource shortages. Similarly, Walker and Gilson (2004) describe how nursing practice is influenced by diverse forces. Nurses are asked to implement policies that might seek to improve patient care, but offer scant regard for nurses and their welfare. They thus exercise their own discretionary power in the delivery of nursing work and their interactions with patients and this has a strong effect on how policies are enacted.

The rich but still quite small literature that examines the context of policy and practice in LMIC hospitals is instructive. The unavailability of resources in hospitals in poorer countries constrains how staff operate and nurses need to adopt strategies to cope that may not align with idealised notions of nursing, nor evidence-based practices. These explorations of medical work in practice show the complex dynamics that make improvement of health systems challenging in LMIC settings – even when resources are provided, the normative model of practice may prove hard to change. Thus, in understanding how nurses cope with stress, we are drawn to understand and describe nursing practice in context. This approach is in contrast to the dominant areas of the study of nursing stress: burnout and resilience, which tend to focus more on the qualities of individuals, rather than on how nurses collectively cope.

Burnout is a wholly negative term, and is most often treated as a kind of endpoint where those suffering from it become useless or worse to the organisations that employ them (Schaufeli et al., 2009). Where the effects of burnout are studied, this tends to be through the questionnaire answers of frontline practitioners using standardised tools (e.g. Maslach and Jackson, 1981; Santinello, 2007). In many ways, resilience is the other side of the coin to burnout. Resilience is generally thought to mean an ability to deal with stress and heavy workloads but, as with burnout, its definition is contested and its use widespread. Within the nursing literature, resilience is most often seen as a set of abilities and characteristics (Tusaie and Dyer, 2004) to be identified or encouraged as part of a process (Jacelon, 1997) that can help individual nurses to cope with difficult working circumstances. Inevitably, resilience of this type is viewed as a highly positive characteristic and one to be encouraged in positions of management (Jackson and Daly, 2011) or high emotional work (McQueen, 2004).

We find that this binary categorisation of burnout and resilience do not provide an adequate explanation of the responses of nurses in the high-pressure environment of the NBU we studied where *collective* efforts to organise work to mitigate stress were apparent. We were driven by our empirical findings to describe nurses who avoid ‘burnout’ but where the resulting nursing ‘resilience’ could hardly be said to be positive. We are interested not only in the personal characteristics of nurses who manage to cope with high workloads, but also the strategies they collectively adopt to cope and allay suffering within this particularly stressful LMIC context (Gill, 2019).

In seeking to investigate how nurses cope under pressure, we connect with earlier academic studies of nursing that focused on collective methods of reducing anxiety that combine to mount a ‘social defence system’ (Menzies, 1960). Menzies examined student nurses and identified ten ways in which they reduced their anxiety through particular practices. We also follow Davina Allen in aiming to construct an empirically-based understanding of aspects of nursing work that is driven by observation of nursing practice (2004, 2007). We find major similarities between three activities that Menzies identified as part of a social defence system and three ‘bundles’ Allen describes as core components of nursing work. By combining their theoretical insights, we are able to show how the reduction of nursing anxiety plays an important role in many core aspects of nursing work.

Following our Extended Case Method approach, we wish to extend out from our findings that highlight the importance of collective coping at the micro level, towards international policies that have identified nurses as central to the quality of care. If nursing is to be ‘patient centred’, then nursing stress needs to be recognised as a collective problem, manifested in a collective coping response, that leads to the type of routinised, and necessarily partial nursing we describe in the following sections.

### 1.2 National context

In countries such as the UK, even for babies who do not require intensive care, guidelines suggest that there should be 1 nurse for every 2 to 4 sick babies (BAPM, 2001; NANN, 2009) with evidence linking lower nurse ratios to higher mortality in high income settings (BAPM, 2001). In the settings we study, nurse to baby ratios may be as low as 1 nurse to 25 babies: a situation that is common across Kenya (Gathara et al., 2019) and other LMICs (Bradley et al., 2015; Vesel et al., 2013) and likely to remain the case without significant changes in financing (Kinfu et al., 2009).

Recent work related to the study we present here has attempted to assess the availability and quality of inpatient newborn care in hospitals in Nairobi City County across the public, private and not-for-profit sectors (Murphy et al., 2016). There is a huge gap between the need for NBU care in Nairobi County (Murphy et al., 2017) and the provision of high quality, appropriate services. Even when the private, not-for-profit, and public sectors are combined, the current hospital providers only treat an estimated 44% of those who need care while 71% of sick newborns accessing hospital care are treated in just four public facilities, putting tremendous stress on these important wards that tend to serve the poorest mothers and their children (Murphy et al., 2018a). Hence, given the importance of these few facilities to a population of over 4 million people, it is crucial that we study how nurses cope under such resource constraints.

Complicating the situation further, the nursing profession has been under considerable public scrutiny in recent years. A series of medical events and related newspaper articles led to scandals that put pressure on Nairobi's nurses (e.g. Daily Nation, 2016). Additionally, disputes over pay and contracts led to healthcare worker strikes around the country, and ultimately led to a nationwide strike after our data collection was completed (Irimu et al., 2018). As such, the resource constraints and high workload were combined with public events that put further pressure on Nairobi's nurses.

## 2 Methods

Ethnography has the power to shine a light on practices that affect the quality of care, which are not easily explored by other means (Reynolds and Lange, 2019). Ethnographic methods allow us to see hospitals as more than ‘identical clones of a global biomedical model’ (Van Der Geest and Finkler, 2004, p1995) and instead as prisms that reflect the core values and beliefs of the culture in which they are both situated and play an important role. Ethnography thus helps us to understand the local cultures of which hospitals are an important part, but also the professional and organisational cultures that affect workers' practice and the way in which clinical staff relate (Lewin and Reeves, 2011).

With regard to our particular methodological approach, we follow Burawoy's Extended Case Method (ECM) (1998). He advises that the ethnographic observer attempts to ‘dwelling in’ the existing, dominant theory of the phenomena being studied until the moment it fails to help explain the data recorded before extending outwards to new theory and connecting with global themes (Burawoy, 1998, p5). This approach has been used to understand nurses' emotional management (Lopez, 2006). Importantly, Burawoy argues for a ‘reflexive’ epistemological position that embraces the subjectivity of the ethnographer. In embracing the subjectivity of our ECM approach, we aim not to ‘write out’ the observer. We use the first person, we use emotional language in describing how people, including ourselves, felt and we reflect on our own field experiences.

### 2.1 Study sites

The nurse interviews and observation were conducted in the newborn units of three public hospitals providing non-tertiary services in Nairobi, Kenya. All hospitals offer inpatient and outpatient services (e.g. immunization, HIV treatment and care and maternity services). Related work to that presented here offers a broad and comprehensive overview of the resource environment of Nairobi's NBUs (Murphy et al., 2018a).

### 2.2 Data collection

Our interactions with the nursing community in Kenya are longstanding, and as health systems researchers, all four authors have been involved in informal discussions, presentations and workshops (Murphy et al., 2018b) regarding neonatal nursing practice before, during and after the period of research. This experience guided and supplemented the research.

Non-participant observation periods covered weekdays and weekends, across all three hospitals' neonatal wards, and across all shifts (250 h in total) and were spread evenly between February 2016 and November 2016. Immersion into the wards happened quickly, and we were very soon witness to discussions, team meetings, disagreements and highly stressful events such as resuscitations and deaths. Extensive field notes were taken that described these events, and also captured the difficulties faced by each researcher in bearing witness, as we have described elsewhere (Jepkosgei et al., 2019).

Interviews were semi-structured and followed an *ethnographic* or *long* approach with the aim of invoking narratives rather than answers (McCracken, 1988) and lasted 1–1.5 h. Interviews were carried out by the first (male), second (female), and third (female) authors. From these narratives, clarifying questions were developed to iteratively check and extend the theory being developed (Burawoy, 1998).

The study began in January, 2015) with 10 interviews with senior stakeholders who were identified as being important leaders in the space (see Table 1). These interviews helped us to define initial semi-structured interview guides and to establish important relationships with nurse leaders that we still maintain. During the second phase (February 2016–November 2016) we then interviewed nurses who had worked in the newborn unit. This included support staff, nurse managers (in-charges) and frontline nurses, but in one setting, the second and third authors conducted two focus group discussions with nurse students (whose clinical rotations within the NBU typically last 2–6weeks). There was only one male nurse in the three locations and so gender was not an important methodological issue. With regard to age, our respondents were on average 37 years of age, which reflected the average across the NBUs. We conducted 22 interviews with hospital staff across all 3 hospitals, with our 17 full-time nurse interviews representing 39% of the NBU nursing workforce in Nairobi. No repeat interviews were carried out.

**Table 1:** Study sample size showing number of interviews in each hospital, cadre of health workers and details of stakeholders interviewed.

Hospital	Nurses	Support staff	Students	Stakeholders (n = 10)
**1**	6	1		Ministry of Health, Nairobi City County Health Team, Nursing Council of Kenya, National Nurses Association of Kenya, Kenya National Union of Nurses, Kenya Medical Training College, Kenya Paediatric Association and the Kenya Medical Association.
**2**	8	1	2 Focus Group Discussions	
**3**	3	1		
**TOTAL**	**17**	**3**	**2**	

### 2.3 Team

The first three authors were involved in the collection of data – both interviews and non-participant observation. The fourth author oversaw the research and assisted in the design of the research, the analysis of data and the development of theory.

### 2.4 Coding

Interviews were transcribed by the third author, and both transcripts and field note data imported into NVivo 10 qualitative software as a shared project. The full team then agreed on a second order set of codes for the next round of analysis. These themes were refined during the research by modifying the interview guide to probe emerging theory, and by relating it to known theories in the literature (Burawoy, 1998).

## 3 Ethics

Ethics approval for the work was given by the Kenyan Medical Research Institute's (KEMRI) Scientific and Ethical Review Unit. We were introduced to our interviewees by senior members of staff with whom we had discussed the research and the background of the research and our aims were explained before each interview. Informed written consent was secured for all interviewees and considerable care was taken to ensure the research did not interrupt nursing work. All were offered the chance to refuse the interview, but none took this option. Transcripts were not returned to interviewees, but we have continued research in this space, and have presented our findings at two of the three hospital sites and at various nursing events in Kenya. We were, however, faced with significant ethical questions during the research that required changes in our approach to data collection (Jepkosgei et al., 2019).

## 4 Results

We first describe the context of the NBUs where the work was conducted providing details regarding the built environment, the other healthcare workers operating in the NBU, the shift patterns and general pattern of work, and the babies themselves. We then draw on Menzies' and Allen's work to describe three areas of nursing activity which are core to the nursing profession, but conducted in such a way as to reduce nursing stress. We then describe two further collective methods nurses use to reduce stress not described in the literature.

### 4.1 The context of nursing practice

The newborn units in our study sites occupied very different spaces with ward layout varying widely. None were purpose-built and all were under-equipped.

For all three hospitals, the NBUs were based across multiple rooms, each separating extremity of need. This category system has three levels, with ‘A’ being the most at-risk infants, often needing oxygen and very close care. Category ‘B’ infants were judged to be stable, with progress assumed to be likely if the treatment plan was maintained. Category ‘C’ patients, whereas, were assumed to be recovering, but perhaps with a need to continue some form of treatment. Patients in this category may, for example, be: waiting to reach a body weight of 2 kgs before they are allowed to leave the hospital; continuing kangaroo care; or sometimes they are simply waiting for their mothers to recover (Murphy et al., 2018b).

Heating was an issue, and nurses struggled to ensure that the temperature was kept high enough for newborns. There was a general shortage of scales, oxygen machines and other equipment. Infection control measures were extremely limited: floors became visibly dirty almost as soon as they were cleaned, tiles were smashed, and functional sinks were too few. The wards were simply not prepared for the numbers of newborns they were commonly dealing with, meaning babies of different mothers often shared cots and incubators.

There were many other professionals among the staff providing care. One of the most important groups working in NBUs are the student nurses. Although unpaid, these students need to complete significant periods of ‘training’ in the NBUs and were often called upon to share the workload.

The paediatricians, doctors and clinical officers often visited the wards and made quiet progress on medical rounds or discussed cases with nurses. They seemed apart from the general management of the NBUs on all sites however, and over the course of the study, it seemed they were on a different orbit to that of the nurses with whom they interacted formally when queries or emergencies arose. Medical work in the NBU environment appeared to our research team to be very much allotted by profession and not contested, potentially in contrast to other nursing sites (Allen, 2002).

Although our research did not involve speaking to mothers or family members, the importance of their role in providing care was obvious. The mothers who used the public health system were poorer, reflecting the fact that those with money would generally opt for the ‘missionary’ or ‘private’ sectors. Additionally, a large portion of the mothers we saw seemed to be young, and often lacked support from family.

The maternity wards were not positioned so as to make regular feeding convenient, and mothers who were themselves patients and often recovering from operations, were expected to make the walk to the NBU on time. We saw nurses offering instruction and orientation to new mothers, on the wards where they had time, such as helping them with feeding and ‘top-tailing’ (cleaning the baby). On a quiet afternoon in one facility, we saw a senior nurse make significant efforts to help a young, distressed mother who had been struggling to bond with her child. Thus, where time allowed, some nurses were able to offer kind and maternal support to individuals.

Of course, the pressure on facilities was acute. Access was restricted to narrow lanes past doctors doing rounds, through ad-hoc arrangements of plastic seats used by breast-feeding mothers, and in between desks and other equipment. It was hard not to dehumanise these children in an environment where they were each bundled up tightly, pressed together and largely anonymous, but we saw them handled with love by their parents, and often with considerable care and affection by nursing staff.

**Fig. 1:**
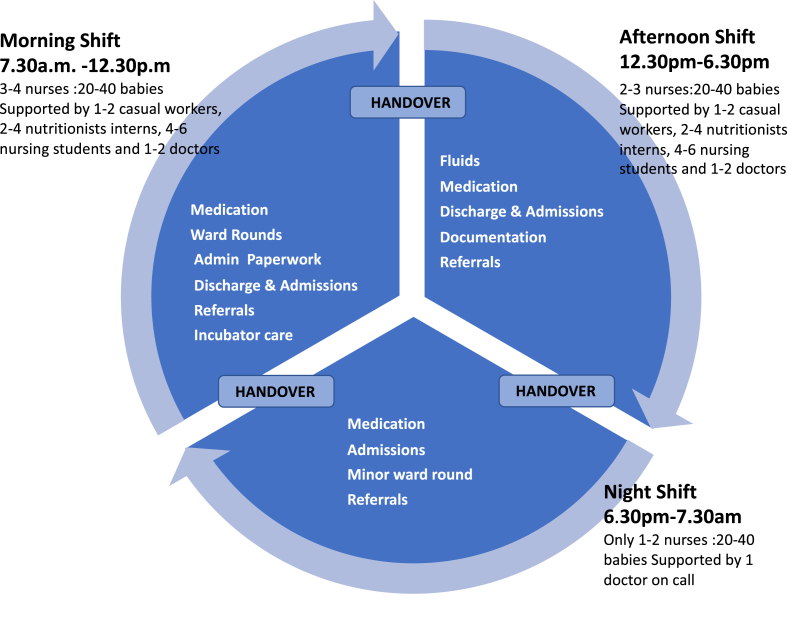
Nursing shifts and activities in Nairobi's NBUs. Modified from Nzinga et al. (2019).

With regard to the organisation of nursing work, a similar shift pattern was observed at all three hospitals. A morning shift, running from 7am to 12:30pm, an afternoon shift from 12:30pm–6pm and a night shift starting at 6pm and concluding at 7:30am. While nursing routines are similar across all phases, each shift has different characteristics and norms. Understanding these differences and the intersections between shifts is important to the overall understanding of Nairobi's NBUs. We have explored the pattern of shifts and its importance to the delivery of care elsewhere (Nzinga et al., 2019), but Fig. 1 highlights the key differences across the shifts.

### 4.2 Practical defensive techniques: routinisation, categorisation, shift patterns, flexibility and pragmatism

“It is automatic – nursing is a routine so everyone who is on duty knows what to do in case the in charge is not around. It is not new – in every ward you go that is the routine”

NBU Nurse

*Routinisation:* The backbone of the working day, and of the template of care that forms around it, is the series of ordered tasks that are associated with each shift (see Fig. 1). The handover, weighing, top-tailing, feeding, provision of medication as a ‘round’, and general monitoring or ‘management by walking around’ as it was termed at one of our sites, are carried out in well-established sequences for each different shift. Our observations led us to recognise that this routine provided a standard route for the nurses to follow through what might otherwise be an unbearably chaotic workplace. Routines similar to those we observed were described by Menzies as serving to help nurses cope (Menzies, 1960). This structure did not appear to be based on any Kenyan policy document, but we noted that it was crucial to the organisation of each shift and was clearly formed normatively, over time and not according to strictly clinical logics. Without the shared, but informal set routines of each shift, nurses would be left to answer the extremely taxing question of how to prioritise time and resources in an environment in which this could never be confidently resolved. Hence, the tasks associated with each shift form a template which is easy to pass on to new staff and gives nurses a start, middle and end that allows them a sense of completion and lets them feel they have ‘done their part’.

“So the number is very large like for today we had seventy babies, we received seventy babies, sometimes you can go up to ninety, a hundred, and then also it depends with the condition of the babies. You might be having very, very critical babies even you can't be able to move out, you go one second then you come back because you know that baby can collapse anytime because that baby needs resuscitation, yeah”

#### NBU nurse

*Shifts* – The logic of the shift timings is hard to fathom without reference to Menzies' theory of the reduction of nurse anxiety (Menzies, 1960). The morning shift is by far the busiest, with the majority of clinical and ‘routine’ work happening during this shift. The afternoon shift, though the same length as the morning shift, is much slower paced. Feeding and continuation of set treatments are the only routine tasks undertaken and the fewer staff on the shift reflect this less onerous design. The night shift is by far the longest at 14 h and there tend to be fewer nurses covering this much longer shift with less support.

According to the nurses, the shifts are arranged this way due to security fears about staff travelling during the dark. These concerns are very genuine, but our observations suggest this arrangement of work also suits nurses for other reasons. The morning shift is loaded with work, but better staffed, meaning that while it is intense, nurses' exposure to stress is reduced due to shared responsibility and the availability of support staff. Many fewer tasks are allocated to the afternoon shift, and the expectation of nurses working afternoon shifts is lower, again reducing nurse suffering but potentially leading to missed care (Blackman et al., 2015). Both morning and afternoon shifts are only 5 h long, meaning that a nurse still has time to travel and complete other tasks in their personal lives.

Planned nursing activity is at its lowest during the night shift, with activity focused on monitoring and maintenance and nurses take significant ‘rest’ periods where possible. Cover from other wards is much reduced too as they are all equally short-staffed, while none of the normal ‘helpers’ are available: nutritionists, students and cleaners do not work nights. Finally, the mothers also tend to sleep, and the nurses are unlikely to remind them to visit the ward to feed their babies due to their reduced working expectations, and so the babies are more likely to skip feeds during night shift. Hence, while the night shift is extremely long and poorly staffed, the expectation of what nurses achieve during this shift drops accordingly, with nursing norms allowing a much lower workload. Nurses spoke about ‘surviving’ nightshifts however, because even though the expected routine work is much lower, they are vulnerable to emergencies where they will have fewer resources available than on the other shifts:

… in the afternoon or maybe late in the night, those are the shifts that are a bit hectic, because in the morning shift maybe because there are students there. Let me give an example of at night – that one is the one you can get the hectic job here. You have received a baby who is bad shape, severe birth asphyxia – this baby needs resuscitation. [This is] a shift [where] you are alone at night, most probably we do one night, one [nurse] per night, so here you have a baby, you are receiving a baby who has come with severe birth asphyxia. When you are dealing with this baby, you still get another baby who the mother has fed and the baby has aspirated. You are the one who is expected to go there and do the first aid, at the same time, this baby might need to be transferred to the next hospital that is Kenyatta. It's you who is to transfer, so all these tasks are with one person. So you get tied up: ‘Do I leave this one? Do I go to Kenyatta?’. So there you are now forced to inform the person who is covering the hospital to give you an assistant, but you see now your head is already worked up, so it becomes hectic. But all in all most of the time you find that you have succeeded in all, so that you will be the first to report in the morning to the in charge; jana mambo haikua poa, lazima atajua hiyo [‘yesterday, things were not good’. He must know that]. But at the end of the day you feel good that those babies didn't die, didn't get anything. You have finished your tasks you have succeeded and you see your nursing is okay.

#### NBU nurse

Through observation of what nurses did and the relative numbers of nurses on each shift, it became clear to us that the arrangement of work across shifts was not explicable in terms of best practice, nor evidence-based guidelines. Instead, it accords with Menzies' theory of collective stress reduction, so that, under normal conditions, the reduced expectations of the afternoon and evening shifts helped nurses to cope. Unfortunately, where emergencies arise on these lower-intensity but less-resourced shifts, nurses are left with even greater loads.

*Categorisation* – On one of the larger wards, a nurse explained that the total number of infants may vary between 40 and 100, but almost as important was the number of high-dependency category ‘A’ babies. These babies require much closer attention, and their demands for more regular medication, monitoring and support, make it harder to manage the other less serious cases. While category ‘B’ babies may also need a lot of care, this triage system means that the concentration of close nursing effort is directed towards a limited number of babies, thus limiting very close and highly emotional exposure to a larger number of infants. The categorisation of infants into the three groups (A, B and C) prioritises very ill children, and it is almost certainly correct that such systems prioritise the sickest babies. The focus on the few however, reduces nurses' exposure to the needs, and the associated emotional burden, of the many who are treated according to the routinised nursing described above. Gathara et al. (2019) measured this effect in a study of the same context, and show both that the Category A babies receive a higher percentage of care than those in B or C. Additionally, they show that lesser staffing (such as that witnessed in afternoon and night shifts) also correlates with a higher percentage of missed nursing care.

*Maintaining the Nursing Kardex –* The Kardex is a simple hand-written record of the nursing treatment given to each patient on the NBU that is updated at the end of each shift. In many of the countries where Kardexs were in use as a medical notation system, it has largely been replaced by Electronic Medical Records (EMR), ‘care-paths’ or integrated into the patient's other medical notes (Newell, 1996). The Kardex is ostensibly a medical document with a wholly rational, practical purpose, but over time, it became clear that in Kenya, nurses' dealings with the Kardex served other less obvious purposes.

“Because no matter how much I'm overwhelmed I'll not hand over kardex to somebody else; I'll have to do it.”

#### NBU nurse

The nurses we saw carried personal notes that allowed them to keep track of patients. Some nurses kept very neat notebooks while others scrawled notes on scraps of paper (A much discussed practice: See Holly and Poletick, 2014). It was these personal notes that nurses used to keep track of patients, and also to record information during handover rather than the Kardex itself. In contrast, the Kardex was kept as a formal document that was updated towards the end of shift as a discrete task alongside the updating of the medical notes. Despite there seeming to be considerable overlap between the medical notes and the Kardex, our suggestions that they might be combined were not well received.

“… sometimes like when I was in paediatrics, sometimes the shift ends when you have not written but like for the critically ill sick patients we have to report because just in case anything happens to them and you did not document, then it will be you did not do anything. So the critically ill patients, but here I have not seen that scenario where we don't document. It's crazy yes, but you have to make sure you document at the end of the day. Even if you are going to take an extra hour or two here, you make sure you have done your part”

#### NBU nurse

The notion that updating and upholding the Kardex is ‘doing your part’ is important here. The management of the Kardex is a shared responsibility between nurses and protects them collectively from criticism from doctors or management. Nurses told us ‘if it's not documented, it's not done’, and also used the Kardex to note issues such as doctors not arriving despite having been called. Hence, it was clear that the Kardex operated as a medico-legal document that, if kept updated, could protect nurses from accusation. This helps to explain why nurses treated the Kardex with such care and made time to ensure that it was fully completed at the end of each shift.

*Flexibility and Autonomy* – The nurses demonstrated a significant degree of flexibility in their dealings with each other. While it was certainly not standard practice, nurses who arrived late for shifts were not punished beyond perhaps being asked to do a less desirable job such as accompanying referrals to other hospitals. Instead, nurses seemed to accept the external pressures in each other's lives. The nurses we witnessed arriving late were not chastised by their colleagues, and nurses asked to stay on to cover a shift until their replacement arrived did so without complaint. This flexibility and understanding among nurses thus ameliorated the external stresses on nursing work such as home life and commuting. However, this behaviour presented a challenge to the organisation of nursing work where it meant that nurses felt they could arrive late for a shift:

“there in the private sector, you are supposed to report at this time, people report on time, everything is done organized – those are the challenges that we face here [in the public sector].”

Additionally, if a nurse was late in starting a shift, it did not necessarily mean that a nurse stayed on shift to cover, but often rather that the shift began with a nurse fewer than expected. The flexibility and autonomy that nurses experienced in the public sector also had a positive side in that it made nurses feel as if their work was less transactional. This less formal and less strict environment was noted as a benefit of the public sector:

“Then again the other feeling when you are here in the public, the difference now between the private and public – the clients; the people you are dealing with. You can identify with them more than the patients in the private sector by the way, so I feel I have done my part … when dealing with the babies and the mothers here, I identify with them. I feel I have helped them. There is not that connection in the private sector because you are dealing with people of, maybe, another class”.

#### NBU nurse

The feeling of doing meaningful work is well known to alleviate stress for nurses (Bargagliotti, 2012), and the nurse's satisfaction in dealing with poorer women from a lower class seemed especially important in this context.

The NBUs were also isolated in terms of their position within the hospital leading to a sense of autonomy and agency which is also known to relieve nurses' stress (Finn, 2001). This meant that there is little external oversight of nursing practice, nor managerial influence over hours and HR issues. This could in part be due to the status of the NBU's patients. Working in the NBU with very small babies was seen as being strongly vocational, requiring a particular calling, again contributing to the meaningfulness of their work:

“… there are people who can't handle the babies and that is why you find if you force people to work in the newborn unit and they don't enjoy, the place will be chaotic.”

#### NBU nurse

Relatedly, there was a feeling that the babies were seen as marginal lives relative to other patients, and this served to distance the work of the NBU from the rest of the hospital:

“And that again is reflected, if they die very quickly then they have the same inpatient number as the mother and the same name as the mother, so they are non-entity if you like.”

“So culturally, we don't even do audits, we don't even do mortality audits when a newborn baby dies. But we do audits, serious audits when a mother dies, serious audits.”

#### Senior nursing stakeholder

The marginality of the infants in the NBU meant that there was perhaps lower societal expectation of survival. Though this area is certainly worth more careful consideration as a research topic, we felt that this cultural facet could indicate that the NBUs were affected by different expectations, either organisational or societal, from those that might be experienced in High Income Countries. We also noted that this sometimes led to nurses framing the likely progress of particular infants in religious and fatalistic terms, with one nurse dealing with a very sick child explaining that ‘in their unit they just keep observing and resuscitating as they wait upon God to do a miracle’.

Finally, there was little external management pressure on the NBUs. The de facto management of the NBUs lay with the nurse ‘in-charge’ and this meant that to a large degree, they had the mandate to organise work as they saw fit. That the nightshift has not been identified as an opportunity for improvement speaks to the lack of clinical oversight in the NBUs we studied and possibly across the whole hospital where this shift pattern prevails. It would be difficult to claim that the acceptance of tardiness and absenteeism was limited to the NBU, but the autonomy that nurses have to organise their own work results in considerable flexibility that allows for accommodation of the busy personal lives of nurses. Though this obviously offers relief to nurses, it also represents a challenge to the running of the wards.

*Pragmatism* – Of course, the sum of formal and routinised nursing tasks does not account for all nursing work in these busy wards. Nurses are constantly faced with new problems to be resolved, emergencies, raw human emotions, mundane administration and frequent mortalities which demand they move beyond their normal routines. Such demands ensure that nurses in this context need to be enormously pragmatic. This pragmatism can manifest in how scarce physical resources are used: we saw nurses using domestic heaters purchased by the medical staff for heating wards and the constant sharing of equipment across wards.

You see, there could be some interdepartmental borrowings like maybe my CPAP or my concentrator has broken down, I need one so urgently. So maybe the paediatric ward they have a very sick baby they want to resuscitate they don't have an Ambu bag they come and borrow ours.

#### NBU nurse

This pragmatism is visible too, in the careful disassembly, cleaning, and re-use of ‘one use’ parts (such as the nasal prongs used for giving oxygen). Nurses are aware of the limitations and risks of such desperate measures, but feel compelled to find workarounds when ultimate responsibility for extremely vulnerable patients lies with them.

“And that prong is such a small thing in the whole circuit such that when the baby uses it, we are supposed to discard the whole system. But we can't afford 40,000[Kenyan Shilling] a baby. So, what we usually take is that little thing and wash it”

#### Senior nurse stakeholder

Such sharing and repurposing gives nurses a sense of ‘personal accomplishment’, and this feeling is known to relieve stress (Maslach and Jackson, 1981).

Similarly, although there is no official ‘task shifting’ or ‘task sharing’ programme in the NBUs we studied, nurses have established a working template of something similar in order to deal with the dearth of formally trained and licensed staff. Mothers were expected to provide the vast majority of the considerable ‘emotional labour’ required in these wards (Allen, 2000), but also to help with the more clinical tasks such as weighing, and feeding of their own babies through nasal-gastric tubes.

“Oh, so that's what we also do, you have to observe as they feed [with nasal -gastric tube], as you take care of your Category A babies, but if you are not comfortable with a mother, you go in and you do it, you show the mother.”

#### NBU nurse

Student nurses and nutritionists could be expected to work long, hard days, regularly being challenged to take on tasks in areas where they had little experience or training. They contrasted this reality of public sector work with the private sector where such behaviour is not allowed. Finally, any and all subordinate staff could expect to be re-directed away from their own daily routines to lend a hand, run errands, direct patients and fetch help. Cleaner ‘casuals’, security staff and porters were all expected to carry out such jobs and we witnessed a strong willingness to do so on their parts. We explore the detail and wider implications of this organic task shifting elsewhere (Nzinga et al., 2019), but here we note that sharing nursing work is an important way to reduce workload and stress.

## 5 Discussion

We explored the setting of Kenyan NBUs in order to understand how nurses cope and how this affects their working practice. Our research asked how nurses cope in this difficult environment, and how this affects nursing practice. Drawing on our ethnography, and the theoretical framing provided by Menzies and Allen, we provide an analysis of the practical, collective strategies nurses deploy for coping with high levels of work and stress.

The first of these, the organisation of patients into particular triage categories helps apportion the limited time and care available, and nurses have been identified as key agents in ‘prioritising care and rationalising resources’ (Allen, 2004, p. 278). In our case however, this is an important method of limiting direct emotional exposure to patients. Nurses need only provide close monitoring with Category A babies, while infants in the other two categories can be dealt with through more distant, sequenced care. Menzies' (1960) views such categorisation as a mechanism of depersonalisation, which in turn, reduces stress on nurses (see Table 2), while Allen (2004) notes that the need to process patients through the cycle of triage is a key function of modern day nursing. The ‘A’, ‘B’, ‘C’ categories work in this regard, but as described above, serve to apportion emotional as well as material resources.

**Table 2:** Empirical findings matched to both key ‘defences’ identified by Menzies and to activities found to be core to the nursing profession by Allen, showing the centrality of these defences to the practices of being a nurse.

Described nursing activity	Components of a defensive system nurses use to reduce anxiety identified by Menzies (1960)	Core bundles of nursing activity identified by Allen (2004, 2007)
A, B, C categorisation	‘Depersonalisation, categorisation and denial of the significance of the individual’, p101	‘Circulating patients’, p274
Routinisation and Shifts	‘Splitting up the nurse-patient relationship’, p101	‘Bringing the individual in’, p274
Maintaining Kardex	‘Reducing the weight of responsibility in decision making by checks and counter-checks’, p104	‘Maintaining a record’, p277
Flexibility and Autonomy		
Pragmatism		

Routinisation is a well-recognised organising logic in healthcare, but is most often viewed pejoratively. It has come to be seen as inferior to ‘patient-centred’ care (Edwards and Elwyn, 2009), but in this instance as with other intensive medical environments (Mazzotta, 2016), a set sequence helps nurses to cope with the less predictable elements of each shift. Similarly, the timing of the shifts and the spread of work across the shifts clearly allows space for nurses to escape the stress of chaotic or close, personalised care. We believe that ‘patient-centred’ care may be impossible in this context as a result.

We also find parallels with Menzies and Allen regarding paperwork. The nurses we studied put great stock in the nursing Kardex as a medico-legal document that protected them from accusations. By signing over the work of the ward in a formal, ritualised way (Strange, 1996), a counterchecking between nurses provided an opportunity for nurses to mutually protect each other from accusation. This was highly important given the accusations of neglect that have been levelled at Kenyan neonatal nurses in recent years.

In summary, we identify three key areas in our study – routinisation, organisation of patients into triage categories and the administration of records – that are described as core nursing activities by Allen that are also identified as methods of collective stress reduction described by Menzies. In addition to these three key areas however, we find two new forms of collective stress-reducing strategies that appear to be related to this LMIC context.

The flexibility that nurses demonstrated in their dealings with each other and the clear autonomy they had in the day-to-day management of work in the NBU offer another form of collective coping. Others have shown the power that nurses have to enact or ignore work policies in similar contexts (Walker and Gilson, 2004), but we wish to draw attention to the fact that such autonomy can be linked to higher job satisfaction (Finn, 2001). The isolation of the NBU and the minimal managerial oversight in the sector leaves a great deal of autonomy for nurses and they are free to help each other by remaining flexible on delays and absences, potentially reducing further exposure to stress.

Finally, we find that nurses in this context cope through improvisation and a spirit of professional pragmatism. Such ‘making do’ is a noted part of nursing in poorer contexts (Aagard, 2009; Strong, 2017) and the nurses in the NBUs we studied demonstrated significant resourcefulness and inventiveness. The unplanned task-shifting that is a major component of such pragmatism is an important area that we address elsewhere (Nzinga et al., 2019). We know from studies of burnout, that a feeling of not being able to do the job, or a lack of ‘personal accomplishment’ contribute towards burnout (Maslach and Jackson, 1981), and conversely, it has been associated with resilience (Mealer et al., 2012) and it seems that finding pragmatic workarounds helps nurses cope here. Of course, ‘making-do’ in frontline care of very sick children only goes so far, and this improvisation is no replacement for qualified, well-trained staff and appropriate equipment.

When combined, the coping mechanisms described here suggest that more effort should be directed towards understanding how the combined pressures on nurses are mitigated through collective action and exploring the effects of coping mechanisms on care. The study of how stress is mitigated through collective nursing work has been neglected, despite its importance. Rather than replace burnout and resilience however, our work could help expand these areas by pointing to how collective action leads to individual resilience. Menzies in a late interview suggested that her famous paper had been misread and she had not intended it to lead to a focus on addressing the emotional needs of individuals, but rather for work to concentrate on how stress and anxiety are contained and mitigated through nursing practice (Lawlor and Webb, 2009). This is a call that we answer.

## 6 Limitations

We recognise that this is a relatively short ethnography. While ‘rapid’ ethnographies have increasingly found favour in global health (Vindrola-Padros and Vindrola-Padros, 2018) and we far exceed the requirements of that approach, we realise that we have set out to tackle a large topic with a relatively low number of interviews and hours of observation. It is worthy of note however, that the work presented was embedded in a broader programme of research and that all four authors continue their work in this field today with many of the nurses quoted above (English et al., 2019).

We also recognise, in line with our chosen Extended Case Method approach, the subjectivity of our work and the ways in which our presence in this field may have altered what our respondents did and said. We have attempted to be mindful of this and have discussed our experience of conducting ethnography in this challenging environment elsewhere (Jepkosgei et al., 2019).

## 7 Conclusion

By extending the theory of Menzies and Allen, we were able to illuminate the collective coping mechanisms used by nurses to alleviate stress in an acute care setting. Abandoning the prevalent theories of burnout and resilience, we used Menzies' and Allen's theories to identify three ways in which nurses work together to cope with the stresses of their work. We also described two new areas: autonomy and flexibility; and pragmatism, that we believe may be associated with the LMIC setting of our study. Resource shortages, minimal managerial oversight, and the embeddedness of nursing work in local culture create both a need and an opportunity for nursing professionals to behave in ways that would not be required nor allowed in richer countries.

We hope to reinvigorate research on the use of collective coping, and in particular, the way that the need to reduce stress influences nursing work normatively over time. The coping mechanisms we discuss are not described by local or national policies and they are not part of nursing curricula. Instead they are indicative of how nurses will tend to work together to alleviate stress, even where this may have implications for the quality of care.

It is an aim of the extended case method to extend outwards from the micro-level findings of ethnographies towards larger societal and global forces. We link our work with recent efforts to highlight the difficulties that nurses face globally (Crisp, 2018), and the importance of their role in providing high quality healthcare (Kruk et al., 2018) and in the NBU in particular (Bhutta et al., 2014). It is timely to carefully study how the now well-recognised overwork of nurses influences the level of care they provide.

Policymakers pursuing quality improvement interventions, particularly those that promote ‘patient centredness’, should recognise several things. Firstly, the need to protect nurses from stress and anxiety is strong and has a significant formative role in the organisation of collective nursing work. Secondly, the subsequent organisation of nursing work may limit the quality of care provided. Thirdly, even if nursing stress is relieved through the increased availability of human and technical resources, the organisation of nursing work has calcified over many decades and will not be easy to change. Finally, the focus on individual burnout and resilience ignores collective coping and may put an improper emphasis on individual nurses to become more personally resilient. Interventions in this area must combine the addition of new human and technical resources with managerial improvements and co-design with nurses if improvements to the quality of care are to be realised.

## Author statement

Jacob McKnight: conceptualisation; methodology; investigation; formal analysis; writing original draft; funding; project admin; Jacinta Nzinga: investigation; writing (review and editing); project admin; formal analysis; Joyline Jepskogei: investigation; writing (review and editing); project admin; formal analysis; Mike English: conceptualisation; writing (review and editing); funding; project admin.

## Appendix A Supplementary data

COREQ checklist Multimedia component 1

## References

[bib1] Aagard M. (2009). Bricolage: making do with what is at hand. Creativ. Nurs..

[bib2] Allen D. (2000). Negotiating the role of expert carers on an adult hospital ward. Sociol. Health Illn..

[bib3] Allen D. (2002). The Changing Shape of Nursing Practice: the Role of Nurses in the Hospital Division of Labour.

[bib4] Allen D. (2004). Re‐reading nursing and re‐writing practice: towards an empirically based reformulation of the nursing mandate. Nurs. Inq..

[bib5] Allen D. (2007). What do you do at work? Profession building and doing nursing. Int. Nurs. Rev..

[bib6] Aluvaala J., Nyamai R., Were F., Wasunna A., Kosgei R., Karumbi J. (2015). Assessment of neonatal care in clinical training facilities in Kenya. Arch. Dis. Child..

[bib7] BAPM (2001). Standards for Hospitals Providing Neonatal Intensive and High Dependency Care.

[bib8] Bargagliotti L.A. (2012). Work engagement in nursing: a concept analysis. J. Adv. Nurs..

[bib9] Bhutta Z.A., Das J.K., Bahl R., Lawn J.E., Salam R.A., Paul V.K. (2014). Can available interventions end preventable deaths in mothers, newborn babies, and stillbirths, and at what cost?. The Lancet.

[bib10] Blackman I., Henderson J., Willis E., Hamilton P., Toffoli L., Verrall C. (2015). Factors influencing why nursing care is missed. J. Clin. Nurs..

[bib11] Bradley S., Kamwendo F., Chipeta E., Chimwaza W., de Pinho H., McAuliffe E. (2015). Too few staff, too many patients: a qualitative study of the impact on obstetric care providers and on quality of care in Malawi. BMC Pregnancy Childbirth.

[bib12] Burawoy M. (1998). The extended case method. Sociol. Theory.

[bib13] Crisp L.N. (2018). Nursing Now – why nurses and midwives will be even more important and influential in the future. Int. Nurs. Rev..

[bib14] d'Alessandro E. (2015). Human activities and microbial geographies. An anthropological approach to the risk of infections in West African hospitals. Soc. Sci. Med..

[bib15] Daily Nation (2016). Give Me My Baby Mother Tells Hospital.

[bib16] Dickson K.E., Simen-Kapeu A., Kinney M.V., Huicho L., Vesel L., Lackritz E. (2014). Every Newborn: health-systems bottlenecks and strategies to accelerate scale-up in countries. Lancet.

[bib17] Duclos D., Faye L., Ndoye T., Penn-Kekana L. (2019). Envisioning, evaluating and co-enacting performance in global health interventions. Anthropol. Action.

[bib18] Edwards A., Elwyn G. (2009). Shared Decision-Making in Health Care: Achieving Evidence-Based Patient Choice.

[bib64] English M., Gathara D., Nzinga J., Kumar P., Were F., Warfa O. (2019). A health policy and systems research programme exploring quality and coverage in Kenya – A summation of findings. BMJ Global Health.

[bib19] Enweronu-Laryea C., Dickson K.E., Moxon S.G., Simen-Kapeu A., Nyange C., Niermeyer S. (2015). Basic newborn care and neonatal resuscitation: a multi-country analysis of health system bottlenecks and potential solutions. BMC Pregnancy Childbirth.

[bib20] Fassin D. (2008). The elementary forms of care: an empirical approach to ethics in a South African Hospital. Soc. Sci. Med..

[bib21] Finn C.P. (2001). Autonomy: an important component for nurses' job satisfaction. Int. J. Nurs. Stud..

[bib22] Gathara D., Opiyo N., Wagai J., Ntoburi S., Ayieko P., Opondo C. (2011). Quality of hospital care for sick newborns and severely malnourished children in Kenya: a two-year descriptive study in 8 hospitals. BMC Health Serv. Res..

[bib23] Gathara D., Serem G., Murphy G.A., Obengo A., Tallam E., Jackson D. (2019). Missed nursing care in newborn units: a cross-sectional direct observational study. BMJ Qual. Saf..

[bib24] Gill M.J. (2019). The significance of suffering in organizations: understanding variation in workers' responses to multiple modes of control. Acad. Manag. Rev..

[bib25] Holly C., Poletick E.B. (2014). A systematic review on the transfer of information during nurse transitions in care. J. Clin. Nurs..

[bib26] Hughes F. (2006). Nurses at the forefront of innovation. Int. Nurs. Rev..

[bib27] Irimu G., Ogero M., Mbevi G., Kariuki C., Gathara D., Akech S. (2018). Tackling health professionals' strikes: an essential part of health system strengthening in Kenya. BMJ Global Health.

[bib28] Jacelon C.S. (1997). The trait and process of resilience. J. Adv. Nurs..

[bib29] Jackson D., Daly J. (2011). All things to all people: adversity and resilience in leadership. Nurse Lead..

[bib30] Jepkosgei J., Nzinga J., Mcknight J. (2019). Maintaining distance and staying immersed: practical ethics in an under-resourced new born unit. J. Empir. Res. Human Res. Ethics.

[bib31] Kinfu Y., Dal Poz M.R., Mercer H., Evans D.B. (2009). The health worker shortage in Africa: are enough physicians and nurses being trained?. Bull. World Health Organ..

[bib32] Kruk M.E., Gage A.D., Arsenault C., Jordan K., Leslie H.H., Roder-DeWan S. (2018). High-quality health systems in the Sustainable Development Goals era: time for a revolution. The Lancet Global Health.

[bib33] Lawlor D., Webb L. (2009). An interview with Isabel Menzies Lyth with a conceptual commentary. Org. Soc. Dyn.: Int. J. Psychoanal. Syst. Group Relat. Perspect..

[bib34] Lawn J.E., Blencowe H., Oza S., You D., Lee A.C.C., Waiswa P. (2014). Progress, priorities, and potential beyond survival. The Lancet.

[bib35] Lewin S., Reeves S. (2011). Enacting ‘team’ and ‘teamwork’: using Goffman's theory of impression management to illuminate interprofessional practice on hospital wards. Soc. Sci. Med..

[bib36] Lopez S.H. (2006). Emotional labor and organized emotional care: conceptualizing nursing home care work. Work Occup..

[bib37] Maslach C., Jackson S.E. (1981). The measurement of experienced burnout. J. Organ. Behav..

[bib38] Mazzotta C.P. (2016). Biomedical approaches to care and their influence on point of care nurses: a scoping review. J. Nurs. Educ. Pract..

[bib39] McCracken G. (1988). The Long Interview.

[bib40] McQueen A.C. (2004). Emotional intelligence in nursing work. J. Adv. Nurs..

[bib41] Mealer M., Jones J., Newman J., McFann K.K., Rothbaum B., Moss M. (2012). The presence of resilience is associated with a healthier psychological profile in intensive care unit (ICU) nurses: results of a national survey. Int. J. Nurs. Stud..

[bib42] Menzies I.E.P. (1960). A case-study in the functioning of social systems as a defence against anxiety:A report on a study of the nursing service of a general hospital. Hum. Relat..

[bib43] Moxon S.G., Lawn J.E., Dickson K.E., Simen-Kapeu A., Gupta G., Deorari A. (2015). Inpatient care of small and sick newborns: a multi-country analysis of health system bottlenecks and potential solutions. BMC Pregnancy Childbirth.

[bib44] Murphy G.A.V., Gathara D., Aluvaala J., Mwachiro J., Abuya N., Ouma P. (2016). Nairobi Newborn Study: a protocol for an observational study to estimate the gaps in provision and quality of inpatient newborn care in Nairobi City County, Kenya. BMJ Open.

[bib45] Murphy G.A.V., Waters D., Ouma P.O., Gathara D., Shepperd S., Snow R.W. (2017). Estimating the need for inpatient neonatal services: an iterative approach employing evidence and expert consensus to guide local policy in Kenya. BMJ Global Health.

[bib46] Murphy G.A.V., Gathara D., Abuya N., Mwachiro J., Ochola S., Ayisi R. (2018). What capacity exists to provide essential inpatient care to small and sick newborns in a high mortality urban setting? - a cross-sectional study in Nairobi City County, Kenya. PLoS One.

[bib47] Murphy G.A.V., Omondi G.B., Gathara D., Abuya N., Mwachiro J., Kuria R. (2018). Expectations for nursing care in newborn units in Kenya: moving from implicit to explicit standards. BMJ Global Health.

[bib48] NANN (2009). Position Statement #3009 - Minimum RN Staffing in NICUs.

[bib49] Newell B.A. (1996). We killed the Kardex so the care path could live. Nurs. Manag..

[bib50] Nzinga Jacinta, McKnight Jacob, Jepkosgei Jepkosgei, English Mike (2019). Exploring the space for task shifting to support nursing on neonatal wards in Kenyan public hospitals. Hum. Resour. Health.

[bib51] Reynolds R., Lange I.L. (2019). Introduction: anthropological knowledge and practice in global health. Anthropol. Action.

[bib52] Santinello M. (2007). LBQ: Link Burnout Questionnaire: Manuale: Giunti OS.

[bib53] Schaufeli W.B., Leiter M.P., Maslach C. (2009). Burnout: 35 years of research and practice. Career Dev. Int..

[bib54] Strange F. (1996). Handover: an ethnographic study of ritual in nursing practice. Intensive Crit. Care Nurs..

[bib55] Strong A.E. (2017). Working in scarcity: effects on social interactions and biomedical care in a Tanzanian hospital. Soc. Sci. Med..

[bib56] Tusaie K., Dyer J. (2004). Resilience: a historical review of the construct. Holist. Nurs. Pract..

[bib57] Van Der Geest S., Finkler K. (2004). Hospital ethnography: introduction. Soc. Sci. Med..

[bib58] Vesel L., Manu A., Lohela T.J., Gabrysch S., Okyere E., ten Asbroek A.H.A., Kirkwood B.R. (2013). Quality of newborn care: a health facility assessment in rural Ghana using survey, vignette and surveillance data. BMJ Open.

[bib59] Vindrola-Padros C., Vindrola-Padros B. (2018). Quick and dirty? A systematic review of the use of rapid ethnographies in healthcare organisation and delivery. BMJ Qual. Saf..

[bib60] Walker L., Gilson L. (2004). ‘We are bitter but we are satisfied’: nurses as street-level bureaucrats in South Africa. Soc. Sci. Med..

[bib61] WHO (2016). Standards for Improving Quality of Maternal and Newborn Care in Health Facilities.

[bib62] WHO (2017). Sustainable Development Goal 3 - Targets and Indicators.

[bib63] Wilson A., Whitaker N., Whitford D. (2012). Rising to the challenge of health care reform with entrepreneurial and intrapreneurial nursing initiatives. Online J. Issues Nurs..

